# Wild Ducks as Long-Distance Vectors of Highly Pathogenic Avian Influenza Virus (H5N1)

**DOI:** 10.3201/eid1404.071016

**Published:** 2008-04

**Authors:** Juthatip Keawcharoen, Debby van Riel, Geert van Amerongen, Theo Bestebroer, Walter E. Beyer, Rob van Lavieren, Albert D.M.E. Osterhaus, Ron A.M. Fouchier, Thijs Kuiken

**Affiliations:** * Erasmus Medical Center, Rotterdam, the Netherlands

**Keywords:** disease vectors, ducks, epidemiology, immunohistochemistry, influenza A virus, H5N1 subtype, influenza in birds, pathology, reverse transcriptase polymerase chain reaction, virology, research

## Abstract

Wild birds have been implicated in the expansion of highly pathogenic avian influenza virus (H5N1) outbreaks across Asia, the Middle East, Europe, and Africa (in addition to traditional transmission by infected poultry, contaminated equipment, and people). Such a role would require wild birds to excrete virus in the absence of debilitating disease. By experimentally infecting wild ducks, we found that tufted ducks, Eurasian pochards, and mallards excreted significantly more virus than common teals, Eurasian wigeons, and gadwalls; yet only tufted ducks and, to a lesser degree, pochards became ill or died. These findings suggest that some wild duck species, particularly mallards, can potentially be long-distance vectors of highly pathogenic avian influenza virus (H5N1) and that others, particularly tufted ducks, are more likely to act as sentinels.

## Abstract 2

Some duck species are potential long-distance vectors; others are more likely to function as sentinels.

The currently ongoing outbreaks caused by highly pathogenic avian influenza virus (HPAIV) of the subtype H5N1 are of concern not only to the poultry industry but also to public health (*1*,*2*). This virus, which causes a high fatality rate among infected patients, may adapt to efficient human-to-human transmission and thus initiate a new human influenza pandemic (*3*). Since 1996, when the ancestor virus was identified in domestic geese from China (*4*), outbreaks have spread and now encompass countries in Asia, the Middle East, Europe, and Africa (*5*). This spread of HPAIV among poultry flocks is traditionally thought to occur by transport of infected poultry, contaminated equipment, and persons associated with the poultry industry (*6*). HPAIV has occasionally been detected in wild birds near affected poultry flocks, but these birds have had limited or no role in virus dissemination (*7*). In the current outbreaks, however, wild birds are suspected of playing a major role as long-distance virus vectors.

During the expansion of HPAI (H5N1) outbreaks from Asia to Europe, 2 events implicated wild birds, particularly waterbirds, as long-distance virus vectors (*8*). First, virus outbreaks in 2005 rapidly spread westward from Russia and Kazakhstan in July and August to Turkey, Romania, and Ukraine in October. Wild waterbirds were suggested as a vector because the virus spread through areas that had no record of any virus presence and coincided with the fall migration of wild waterbirds between these areas. Second, at the beginning of 2006, HPAIV (H5N1) was detected in many wild waterbirds in western Europe, often in areas where no outbreaks had been detected among intensively surveyed poultry (*9*–*12*); this event overlapped with unusual waterbird movements associated with cold weather in the Black Sea area. Quantitative analysis of the global spread of HPAIV (H5N1) also supports the potential role of migratory wild birds in virus spread (*13*). In June and July of 2007, Germany, France, and the Czech Republic again reported HPAIV (H5N1) in wild waterbirds (*14*), which illustrates their ongoing involvement in the epidemiology of this viral infection.

The main argument against the view that wild waterbirds are long-distance vectors of HPAIV (H5N1) is that most wild waterbirds in which this virus was identified were either sick or dead, which suggests that they were too severely affected to spread the virus over any substantial distance (*15*). This argument is supported by experimental evidence that over time HPAIV (H5N1) has become more pathogenic for domestic ducks. Although domestic ducks did not show clinical disease or death from HPAI V (H5N1) isolates from 2001 or before, experimental infection of domestic ducks with an HPAIV (H5N1) isolate from 2002 caused neurologic disease and death (*16*). This high pathogenicity has been shown to be associated with molecular changes in the polymerase genes PA and PB1 (*17*). However, little is known about the pathogenicity and excretion pattern of recent HPAIV (H5N1) isolates in wild waterbird species whose migration patterns correspond with the observed westward expansion of HPAI (H5N1) outbreaks.

To test the hypothesis that wild waterbirds can excrete HPAIV (H5N1) in the absence of debilitating disease and so potentially act as long-distance virus vectors, we experimentally infected 6 species of wild ducks with an avian isolate of HPAIV (H5N1) from Europe, obtained in 2005 (A/turkey/Turkey/1/2005). We chose ducks because they are an important group in the epidemiology of avian influenza in the wild, although other waterbird species, such as geese, swans, and gulls, also play a role (*15*). We chose these particular duck species because of their abundance, preference for freshwater habitats, and migratory pattern spanning Asia, Europe, and Africa (Table; Appendix Figure 1. All 6 species are listed by the European Union as carrying a higher risk for avian influenza (*18*).

**Table:** Health status and virus excretion of 46 wild ducks experimentally infected with highly pathogenic avian influenza virus (H5N1)

Common name (taxonomic name), n = 8 each	No. ducks with clinical signs			No. ducks that excreted virus from*				
				Pharynx			Cloaca	
	Mild	Severe		Virus isolation	RT-PCR		Virus isolation	RT-PCR
Tufted duck (*Aythya fuligula*)†	4	3		6	7		0	5
Eurasian pochard (*Aythya ferina*)†	3	1		7	7		2	5
Mallard (*Anas platyrhynchos*)	0	0		8	8		0	5
Common teal (*Anas crecca*)	0	0		3	7		1	4
Eurasian wigeon (*Anas penelope*)	0	0		4	7		0	0
Gadwall (*Anas strepera*)	0	0		7	8		0	8
Total	7	4		35	44		3	27

*Positive result from any swab collected during the experiment. RT-PCR, reverse transcription–PCR. †One bird removed after inoculation because of concurrent disease.

## Materials and Methods

### Virus Preparation

A virus stock of influenza virus A/turkey/Turkey/1/2005 (H5N1) was prepared by 2 passages in 10-day-old embryonated chicken eggs. The harvested allantoic fluid had a titer (*19*) of 1.3 × 10^8^ median tissue culture infectious dose (TCID_50_)/mL and was diluted with phosphate-buffered saline (PBS) to obtain a final titer of 3.3 × 10^3^ TCID_50_/mL. All experiments with HPAIV (H5N1) were performed under Biosafety Level 3+ conditions.

### Animals

We experimentally infected 6 species of ducks: 2 species of diving ducks belonging to the genus *Aythya* (tufted duck [*A. fuligula*] and Eurasian pochard [*A. ferina*]) and 4 species of dabbling ducks belonging to the genus *Anas* (mallard [*A. platyrhynchos*], common teal [*A. crecca*], Eurasian wigeon [*A. penelope*], and gadwall [*A. strepera*]). The *Anas* species represent 3 clades: the mallard represents the mallard clade, the common teal represents the green-winged teal clade, and the Eurasian wigeon and gadwall represent the wigeon clade, previously belonging to the genus *Strepera* (*20*). For each species, males and females were equally represented. All ducks used for the infection experiments were captive-bred (Dierenhandel Hoogendoorn, Stolwijk, the Netherlands) and housed indoors since hatching to minimize the risk for inadvertent avian influenza virus infection. Birds were 8–11 months of age at time of inoculation. Serum samples, cloacal swabs, and pharyngeal swabs were collected from all ducks 1 week before inoculation. Serum was analyzed by using a commercially available influenza A virus antibody ELISA kit for the detection of antibodies against nucleoprotein (European Veterinary Laboratory, Woerden, the Netherlands) according to the manufacturer’s instructions. Swabs were tested by reverse transcription–PCR (RT-PCR). No duck had antinucleoprotein antibody, except 1 pochard. Its serologic status did not protect it from HPAIV (H5N1) infection; it had the most severe clinical signs of all inoculated pochards and died at 4 days postinoculation (dpi). For 1 teal and 2 pochards, titers were suspected positive. No duck used for the infection experiments was positive for avian influenza virus by RT-PCR. We used 8 specific-pathogen–free White Leghorn chickens, 4–6 weeks old, as controls for the pathogenicity of the virus stock.

### Experimental Design

For each species, 8 birds were housed together in negatively pressurized isolator units. Each bird was inoculated with 1 × 10^4^ TCID_50_ HPAIV (H5N1), 1.5 mL intratracheally and 1.5 mL intraesophageally. We used this low dose to increase the chance of inducing a subclinical infection and to simulate field circumstances. In addition, 4 birds per duck species, which served as negative controls, were sham inoculated in the same manner with PBS-diluted sterile allantoic fluid. Each day, a qualified veterinarian scored clinical signs of disease in all birds according to a standardized list. Cloacal and pharyngeal swabs were collected in 1 mL transport medium (*21*) daily for the first 14 days and every 2 days thereafter.

We randomly divided each group of 8 birds into 2 groups of 4. One group was euthanized by exsanguination under isoflurane anesthesia for pathologic examination at 4 dpi; the other group was monitored for virus excretion until 18–21 dpi. Two ducks were removed after inoculation: 1 tufted duck because of concurrent aspergillosis and 1 pochard because of concurrent staphylococcosis. Also, 1 pochard and 3 tufted ducks were were chosen for pathologic examination at 4 dpi because they were dead or moribund. Although this was not random sampling, it does reflect the field situation because dead ducks no longer actively excrete virus. As expected, by 2 dpi 100% of the positive-control chickens were sick or dead, whereas the negative-control ducks showed no clinical signs and were euthanized at 4 dpi. Animal studies were approved by an independent animal ethics committee and performed under Biosafety Level 3+ conditions.

### Pathologic Examination and Immunohistochemical Testing

Necropsies and tissue sampling were performed according to a standard protocol. After fixation in 10% neutral-buffered formalin and embedding in paraffin, tissue sections were examined by 1 of 2 methods: hematoxylin and eosin staining for histologic evaluation or an immunohistologic method that used a monoclonal antibody against nucleoprotein of influenza A virus as a primary antibody for detection of influenza viral antigen (*22*). The positive control was lung tissue of an HPAIV (H5N1)–infected domestic cat; negative controls were omission of primary antibody, substitution of primary antibody by an irrelevant monoclonal antibody of the same isotype, and testing of tissues from sham-inoculated ducks. The following tissues were examined: brain (cerebrum, cerebellum, brainstem), trachea, bronchus, lung, caudothoracic or abdominal air sac, esophagus, proventriculus, duodenum, pancreas, liver, jejunum, ileum, cecum, colon, bursa of Fabricius, spleen, kidney, gonad (testis or ovary), heart, pectoral muscle, and adrenal gland.

### RT-PCR and Virus Titration

Tissue samples were weighed and homogenized in 3 mL of transport medium by use of a homogenizer (Kinematica Polytron, Lucerne, Switzerland). RNA isolation and RT-PCR were performed as described (*23*). Briefly, RNA from swabs and tissue suspensions was isolated by using a MagNaPure LC system with the MagNaPure LC Total Nucleic Acid Isolation Kit (Roche Diagnostics, Almere, the Netherlands). Real-time RT-PCR assays were performed on an ABI Prism 7000 Sequence Detection System machine (Applied Biosystems, Foster City, CA, USA) by using the TaqMan EZ RT-PCR Core Reagents Kit (Applied Biosystems, Nieuwerkerk a/d IJssel, the Netherlands) according to the manufacturer’s instructions. The test used a hybridization probe (5′-6-FAM-TTT-ATT-CAA-CAG-TGG-CGA-GTT-CCC-TAG-CAC-T-TAMRA-3′) and specified primers (forward: 5′-GAG-AGG-AAA-TAA-GTG-GAG-TAA-AAT-TGG-A-3′ and reverse: 5′-AAG-ATA-GAC-CAG-CTA-CCA-TGA-TTG-C-3′) to detect the hemagglutinin gene of HPAIV (H5N1). For each run the samples were prepared and processed in parallel with several negative and positive control samples. Virus titers were determined by serial 10-fold dilution of the homogenized tissue samples and swabs on MDCK cells, as described (*19*). Virus titrations were performed in quadruplicate.

## Results

**Figure 1:**
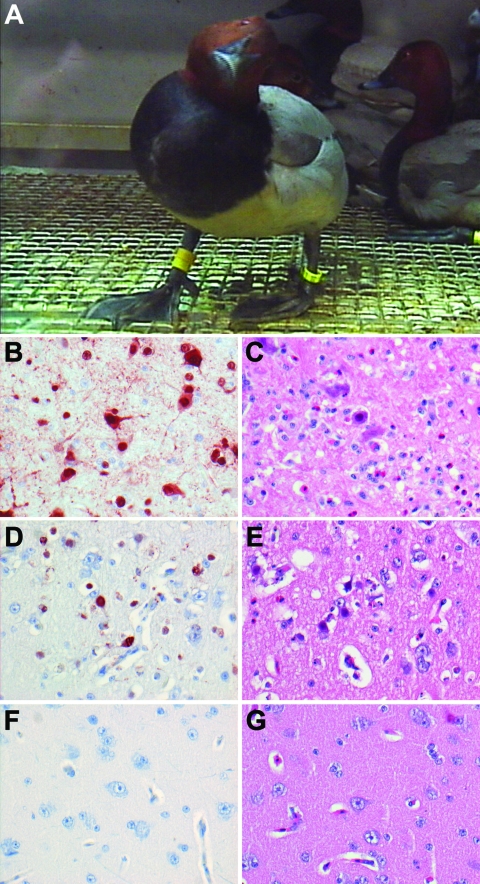
Central nervous system changes in wild ducks experimentally infected with highly pathogenic avian influenza virus (H5N1). A) Torticollis in a pochard. B) Severe multifocal encephalitis, characterized by abundant influenza virus antigen expression in neurons and glial cells and C) extensive necrosis and inflammation, in a tufted duck. D) Rare virus antigen expression in neurons and E) mild necrosis and inflammation in a gadwall that did not show neurologic signs and had only mild focal encephalitis. F) Lack of virus antigen expression and G) lack of necrosis and inflammation in brain tissue of a mallard that did not show neurologic signs. Tissues were stained either by immunohistochemistry that used a monoclonal antibody against the nucleoprotein of influenza A virus as a primary antibody (B, D, F) or with hematoxylin and eosin (C, E, G); original magnification ×100.

Despite the low doses of virus used to inoculate the ducks, rates of productive infection in the 6 species were high: 76% according to virus isolation and 93% according to RT-PCR (Table). HPAIV (H5N1) infection caused clinical signs of disease in only tufted ducks and pochards, both of which are diving ducks in the genus *Aythya* (Table). In contrast, the remaining 4 species—all dabbling ducks belonging to the genus *Anas*—were clinically unaffected. Clinical signs, which were more severe in tufted ducks than in pochards, developed at 3 to 4 dpi and consisted of labored breathing, increased recumbency, and neurologic signs (torticollis [Figure 1, panel **A**], circling, loss of balance, and head tremors). Severely affected birds died or were euthanized in a moribund state at 4 dpi. Mildly affected birds recovered by 7 or 8 dpi.

Severe neurologic signs in tufted ducks and pochards were associated with multifocal viral encephalitis. Although no gross brain lesions were noted (Appendix Table 1, according to laboratory analysis these birds had multiple foci of influenza virus antigen expression (Figure 1, panel **B**; Appendix Table 2, associated with severe necrosis and inflammation (Figure 1, panel **C**) and high virus titers (10^3.5^ to 10^5.2^ TCID_50_ per g tissue) in the brain (Appendix Table 3). The only other ducks with evidence of HPAIV (H5N1) infection of the brain were gadwalls, none of which showed neurologic signs. Gadwalls had only focal influenza virus antigen expression (Figure 1, panel **D**; Appendix Table 2), mild encephalitis (Figure 1, panel **E**), and low virus titers (10^1.5^ TCID_50_ per g tissue) in the brain (Appendix Table 3). No other species had evidence of HPAIV (H5N1) infection in the brain according to immunohistochemical testing, histologic examination, or virus isolation (Figure 1, panels **F** and **G**; Appendix Tables 2 and 3), although individual animals did have positive RT-PCR results for the brain (Appendix Table 4).

**Figure 2:**
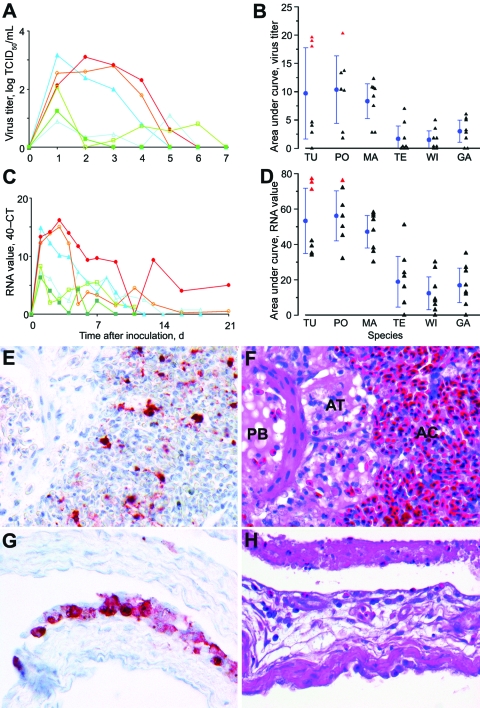
Mean pharyngeal excretion of highly pathogenic avian influenza virus (H5N1) of wild ducks by A) virus isolation and C) reverse transcription–PCR (RT-PCR). Pochard (red, closed circle), tufted duck (orange, open circle), mallard (dark blue, closed triangle), teal (light blue, open triangle), wigeon (dark green, closed square), gadwall (light green, open square); TCID_50_, median tissue culture infectious dose; Ct, cycle threshold. Area under the curve in the first 4 days postinoculation (mean ± 95% confidence interval) for B) virus isolation and D) RT-PCR. TU, tufted duck; PO, pochard; MA, mallard; TE, teal; WI, wigeon; GA, gadwall; red triangles, birds with severe clinical signs; black triangles, birds with mild or no clinical signs. E) Influenza virus antigen expression in epithelial cells in bronchus, parabronchus, atrium, and air capillaries of a tufted duck. F) Bronchointerstitial pneumonia, characterized by flooding of parabronchi (PB), atria (AT), and air capillaries (AC) with proteinaceous fluid and inflammatory cells in a tufted duck. G) Influenza virus antigen expression in epithelial cells lining the air sac wall and H) epithelial necrosis and lymphocytic infiltration in a gadwall. E–H original magnification ×100. Tissues were stained either by immunohistochemistry that used a monoclonal antibody against the nucleoprotein of influenza A virus as a primary antibody (E, G) or with hematoxylin and eosin (F, H).

Pharyngeal excretion of HPAIV (H5N1) varied significantly among the 6 duck species (1-way analysis of variance of area under pharyngeal excretion curve up to 4 dpi, p<0.001), by virus isolation (Figure 2, panel **A**) and by RT-PCR (Figure 2, panel **C**). The ducks could be divided into a high-excretion group consisting of tufted ducks, pochards, and mallards, and a low-excretion group consisting of teals, wigeons, and gadwalls (Figure 2, panels **B** and **D**). Pharyngeal excretion also varied substantially among individuals within species (Appendix Figures 6 and 3, respectively.). This finding was most extreme in tufted ducks and pochards, the species in which the individuals with the highest excretion level were also those showing severe clinical signs (Figure 2, panels **B** and **D**).

Pharyngeally excreted HPAIV (H5N1) likely originated from lung, air sac, or both, because these were the only tissues in the respiratory tract that had immunohistochemical evidence of virus replication (Figure 2, panels **E** and **G**; Appendix Table 2) and because virus was frequently detected in these tissues by virus isolation (Appendix Table 3) and RT-PCR (Appendix Table 4). The histologic lesions corresponding to influenza virus antigen expression in these tissues were bronchointerstitial pneumonia (Figure 2, panel **F**) and lymphocytic airsacculitis (Figure 2, panel **H**). Despite frequent isolation of HPAIV (H5N1) from trachea and extrapulmonary bronchus (Appendix Table 3), these tissues had neither histopathologic nor immunohistochemical evidence of HPAIV (H5N1) replication (Appendix Table 2), which suggests that virus isolated from these sites at 4 dpi originated from elsewhere in the respiratory tract.

**Figure 3:**
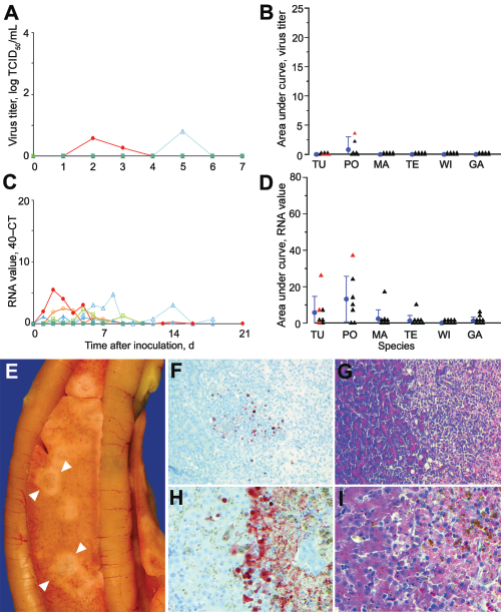
Mean cloacal excretion of highly pathogenic avian influenza virus (H5N1) by wild ducks by A) virus isolation and C) reverse transcription–PCR (RT-PCR). Legend for panels A–D as in Figure 2. E) Pancreas showing multiple foci of necrosis (between arrowheads) in a pochard. F) Pancreatic acinar cells in a pochard and H) hepatocytes in a tufted duck, showing the transition area between normal and necrotic tissue expressing abundant influenza virus antigen. G) Pancreatic lesions in a pochard and I) hepatic lesions in a tufted duck, characterized by sharp transition between normal tissue (left side of panels) and foci of necrosis and inflammatory cell infiltration (right side of panels). F, G original magnification ×50. H, I original magnification ×100. Tissues were stained either by immunohistochemistry that used a monoclonal antibody against the nucleoprotein of influenza A virus as a primary antibody (F, H) or with hematoxylin and eosin (G, I).

Cloacal excretion of HPAIV (H5N1) was uncommon; virus was detected in cloacal swabs of only 7% of ducks by virus isolation and 59% by RT-PCR (Table). Cloacal excretion was exceeded by pharyngeal excretion in all 6 duck species, according to virus isolation (Figure 3, panels **A** and **B**; Appendix Figure 2) and RT-PCR (Figure 3, panels **C** and **D**; Appendix Figure 3).

Cloacally excreted virus likely originated from pancreas, liver, or both, on the basis of the significant association between virus antigen expression in these tissues (Appendix Table 2) and virus detection in cloacal swabs by RT-PCR (Appendix Table 4); 7 of 8 birds with virus antigen expression in liver, pancreas, or both had PCR-positive cloacal swabs between 1 and 4 dpi, in contrast to 0 of 15 birds without virus antigen expression in these tissues (Fisher exact test, p<0.00001). In the pancreas, tufted ducks and pochards had multifocal necrosis (Figure 3, panel **E**), which was the most prominent gross lesion associated with HPAIV (H5N1) infection in this study (Appendix Table 1). Virus antigen expression (Figure 3, panel **F**) occurred at the transition between normal parenchyma and these necrotic foci (Figure 3, panel **G**) and corresponded with high virus titers (10^1.5^ to 10^6.2^ TCID_50_ per g tissue) in the pancreas (Appendix Table 3). In the liver, widespread virus antigen expression (Figure 3, panel **H**) was associated with necrotizing hepatitis (Figure 3, panel **I**) and variable virus titers (no virus isolated to 10^6.2^ TCID_50_ per g tissue) in the liver (Appendix Table 3). Virus produced in pancreas and liver could potentially have reached the intestinal lumen through pancreatic and bile ducts, respectively. Although virus antigen expression was detected in several other tissues, virus originating from these sites likely did not contribute to virus excretion.

It is unlikely that cloacally excreted virus originated from the intestinal, urinary, or genital tracts, although all 3 tracts empty into the cloaca. In the intestine, no virus antigen expression was found in the intestinal epithelium of any of the 23 ducks examined. Virus antigen expression was found in neurons and satellite cells in the peripheral nervous system (submucosal and myenteric plexi, mesenteric ganglia) of the small intestine (Appendix Figure 4, panel **A**), in association with necrotizing ganglioneuritis (Appendix Figure 4, panel **B**), and in myocytes in the lamina muscularis mucosae of the colon, without associated histologic lesions. However, these tissues do not empty into the intestinal lumen. No virus antigen expression was found in tissues of urinary tract (kidney) or genital tract (testis or ovary) (Appendix Table 2). The occasional isolation of HPAIV (H5N1) from kidney samples (Appendix Table 3) may be explained by inadvertent sampling of overlying air sac wall, which did express influenza virus antigen.

Evidence of HPAIV (H5N1) replication was found sporadically in tissues other than those of the respiratory, digestive, and nervous systems (Appendix Table 2). Virus antigen expression was detected in multiple foci of medullary and cortical cells of the adrenal gland (Appendix Figure 4, panel **C**) and was associated with necrotizing adrenalitis (Appendix Figure 4, panel **D**). Virus antigen expression was also detected in multiple foci of myocytes of the heart and was associated with myocardial necrosis.

## Discussion

Our study shows that of the 6 wild duck species studied, the mallard is the prime candidate for being a long-distance vector of HPAIV (H5N1) because it was the only species to show abundant virus excretion without clinical or pathologic evidence of debilitating disease (Table; Figure 2, panels **B** and **D**). These findings fit with the absence of dead mallards in wild bird die-offs from HPAIV (H5N1) in Europe and Asia in 2005 and 2006 (*14*,*24*,*25*), although HPAIV (H5N1) was detected in 1 dead mallard during the recent 2007 HPAI (H5N1) outbreak in wild birds in Germany (*26*). Other characteristics of the mallard support its potential role as a vector ([*27*]*;* Appendix Figure 1): it is the most abundant anatid species in Western Eurasia (≈9 million birds); part of the population migrates long distances northeast to southwest between breeding and wintering areas; and it is found on nearly every type of wetland and is very tolerant of human presence, thus forming a potential link between wild waterfowl, domestic animals, and humans.

Pochards and tufted ducks are less likely candidates as long-distance virus vectors because those individuals that excreted the most virus also developed severe neurologic disease (Figure 2, panels **B** and **D**) and therefore would not have been able to fly far before succumbing. Instead, they are more likely to act as sentinels for HPAIV (H5N1) in wild bird populations, as do mute swans (*Cygnus olor*) (*9*). However, pochards cannot be ruled out as potential vectors because some birds excreted abundant virus in absence of severe clinical signs (Figure 2, panels **B** and **D**). Our results correspond with field observations of pochards and tufted ducks involved in wild bird die-offs from HPAIV (H5N1) infection in France, Germany, and Sweden early in 2006 (*14*). Some of these birds showed clinical signs of neurologic disease, e.g., compulsively swimming around in circles (*28*). Therefore, close surveillance of these 2 *Aythya* species for unusual illness, particularly neurologic disease, or death should provide early warning for HPAIV (H5N1) infection in an area. Redheads (*Aythya americana*), which are diving ducks indigenous to North America, experimentally infected with a 2005 isolate of HPAIV (H5N1) neither showed clinical signs nor died (*29*). Of the 6 species tested, the 3 remaining *Anas* species—gadwall, teal, and wigeon—are the least likely candidates as long-distance virus vectors because they had limited virus excretion (Figure 2, panels **B** and **D**).

HPAIV (H5N1) infection in these wild ducks contrasts in pattern of excretion with that of low-pathogenicity avian influenza virus infection in wild ducks and contrasts in pattern of disease with that of HPAIV infection in chickens. Both contrasts can be explained by the specific tissue tropism of HPAIV (H5N1) in wild ducks. With regard to pattern of excretion, low cloacal excretion was associated with lack of evidence for HPAIV (H5N1) replication in intestinal epithelium of any of the 23 ducks examined (Appendix Table 2), in contrast to most low-pathogenicity avian influenza viruses for which intestine is the main replication site (*30*). Instead, HPAIV (H5N1) replicated preferentially in the respiratory tract (Appendix Tables 2 and 4), which corresponds with high pharyngeal excretion. How this preferential pharyngeal excretion of HPAIV (H5N1) affects its spread and persistence in a wild duck population remains to be determined.

Severe clinical disease in the HPAIV (H5N1)–infected tufted ducks and pochards manifested itself mainly as neurologic signs at about 4 dpi, although pathologic examination also showed virus-induced lesions in organs other than the brain. These findings differ substantially from those of HPAIV (H5N1)–infected chickens, which are characterized mainly by widespread hemorrhage and edema and death by about 2 dpi (*31*). Again, this contrast can be explained by differences in tissue tropism. Whereas the cardiovascular lesions in poultry are associated with widespread replication of HPAIV (H5N1) in endothelium lining the blood vessels (*31*), no such endotheliotropism was detected in any of 23 ducks examined.

The knowledge gained from this study has several implications for surveillance in wild ducks. Active surveillance (sampling of apparently healthy wild birds) should give priority to mallards and, to a lesser degree, pochards. Sampling should not be limited to cloacal swabs, as is the custom in surveillance for low-pathogenicity avian influenza virus, but should include pharyngeal swabs. Passive surveillance (sampling of diseased or dead birds), should pay extra attention to tufted ducks and pochards, particularly those exhibiting neurologic disease. Sampling of wild duck carcasses should not be limited to cloacal, pharyngeal, and tracheal swabs and should include internal organs such as brain, trachea, lung, pancreas, liver, kidney, and spleen (Appendix Tables 3 and 4).

## Supplementary Material

Appendix Table 1 Gross lesions in wild ducks at 4 days postinoculation with highly pathogenic avian influenza virus (H5N1)

Appendix Table 2 Expression of antigen in organs of wild ducks at 4 days postinoculation with highly pathogenic avian influenza virus (H5N1)

Appendix Table 3 Isolation of highly pathogenic avian influenza virus (H5N1) from organs of wild ducks at 4 days after inoculation and from swabs at 1-4 days postinoculation

Appendix Table 4 Detection of highly pathogenic avian influenza virus (H5N1) by reverse transcription-PCR from organs of wild ducks at 4 days postinoculation and from swabs at 1-4 days postinoculation

Appendix Figure 1 Distribution in the eastern hemisphere of the 6 wild duck species used in this study. Yellow, summer (breeding) range; blue: winter range; green, permanent range. (Sources: del Hoyo J, Elliot A, Sargatal J, editors. Handbook of the birds of the world. Volume 1: Ostrich to ducks. Barcelona: Lynx Edicions, 1992; Mullarney K, Svensson L, Zetterström D, Grant PJ. ANWB bird guide of Europe [in Dutch]. Baarn, the Netherlands: Tirion Uitgevers, 2000.)

Appendix Figure 2 Individual pharyngeal (A) and cloacal (B) excretion of highly pathogenic avian influenza virus (H5N1) in wild duck species, by virus isolation.

Appendix Figure 3 Individual pharyngeal (A) and cloacal (B) excretion of highly pathogenic avian influenza virus (H5N1) in wild duck species, by reverse transcription-PCR.

Appendix Figure 4 Highly pathogenic avian influenza virus (H5N1) infection in tufted ducks. A) Neurons and satellite cells in a mesenteric ganglion expressing abundant influenza virus antigen. B) Ganglioneuritis in the same mesenteric ganglion, characterized by neuronal necrosis (arrows) and lymphocyte infiltration (between arrowheads). C) Expression of influenza virus antigen in medullary and cortical cells of an adrenal gland and D) focal necrosis, characterized by hypereosinophilia, pyknosis, and vacuolization (between arrowheads). Original magnification ×100. Tissues were stained either by immunohistochemistry that used a monoclonal antibody against the nucleoprotein of influenza A virus as a primary antibody (A, C) or with hematoxylin and eosin (B, D).
