# Supplementary material for: Wild Ducks as Long-Distance Vectors of Highly Pathogenic Avian Influenza Virus (H5N1)
Source: Emerg Infect Dis. 2008 Apr;14(4):600–7. doi: 10.3201/eid1404.071016 (PMC2570914; doi:10.3201/eid1404.071016)
Supplement: Appendix Table 1 — Gross lesions in wild ducks at 4 days postinoculation with highly pathogenic avian influenza virus (H5N1) [file 07-1016_appT1.pdf]

Appendix Table 1. Gross lesions in wild ducks at 4 days postinoculation with highly pathogenic avian influenza virus (H5N1)

	Gross lesions per species*																					
	Tufted duck no.			Pochard no.				Mallard no.				Teal no.				Wigeon no.				Gadwall no.			
Organ†	1	2	3	1	2	3	4	1	2	3	4	1	2	3	4	1	2	3	4	1	2	3	4
Air sac‡		yellow	yellow	yellow	yellow		yellow	yellow	yellow	yellow	yellow					yellow				yellow		yellow	
Lung§			yellow																	yellow			
Pancreas¶		orange	orange			yellow	orange																
Liver#	red																						

*Severity of lesion: white, no visible lesion; yellow, mild; orange, moderate; red, marked.
†No gross lesions were observed in other organs.
‡Airsacculitis, characterized by mild, multifocal to diffuse opacity and thickening of the air sacs.
§Multifocal pulmonary consolidation.
¶Multifocal pancreatic necrosis, consisting of red or gray foci of 1–4 mm in diameter affecting up to 25% of the pancreas.
#Diffuse hepatic necrosis.
