# Supplementary material for: Wild Ducks as Long-Distance Vectors of Highly Pathogenic Avian Influenza Virus (H5N1)
Source: Emerg Infect Dis. 2008 Apr;14(4):600–7. doi: 10.3201/eid1404.071016 (PMC2570914; doi:10.3201/eid1404.071016)
Supplement: Appendix Table 2 — Expression of antigen in organs of wild ducks at 4 days postinoculation with highly pathogenic avian influenza virus (H5N1) [file 07-1016_appT2.pdf]

Appendix Table 2. Expression of antigen in organs of wild ducks at 4 days postinoculation with highly pathogenic avian influenza virus (H5N1)

		Antigen expression per species*																					
		Tufted duck no.			Pochard no.				Mallard no.				Teal no.				Wigeon no.				Gadwall no.			
System	Organ or tissue†	1	2	3	1	2	3	4	1	2	3	4	1	2	3	4	1	2	3	4	1	2	3	4
Nervous	Brain	red	red	red			red															yellow	yellow	
	Peripheral nerve	orange	red	yellow			yellow																	
Respiratory	Lung		yellow	orange					‡	‡	‡	‡									yellow			
	Air sac	yellow	‡	yellow	‡	‡	orange	‡	‡	‡	‡	‡	‡	‡	‡	‡	‡	‡	‡	‡	‡	yellow	‡	‡
Digestive	Pancreas	red	yellow	red			red	yellow								‡						yellow	yellow	
	Liver	red			yellow		orange															yellow		
	Colon																					yellow		
Other	Heart	orange					yellow																	
	Adrenal gland	red					orange															yellow		

*White, no cells positive; yellow, rare cells positive; orange, moderate number of cells positive; red, many cells positive.
†No virus antigen expression was observed in other organs or tissues.
‡Presence of histologic lesions consistent with infection but no viral antigen expression.
