# Supplementary material for: Wild Ducks as Long-Distance Vectors of Highly Pathogenic Avian Influenza Virus (H5N1)
Source: Emerg Infect Dis. 2008 Apr;14(4):600–7. doi: 10.3201/eid1404.071016 (PMC2570914; doi:10.3201/eid1404.071016)
Supplement: Appendix Table 3 — Isolation of highly pathogenic avian influenza virus (H5N1) from organs of wild ducks at 4 days after inoculation and from swabs at 1-4 days postinoculation [file 07-1016_appT3.pdf]

Appendix Table 3. Isolation of highly pathogenic avian influenza virus (H5N1) from organs of wild ducks at 4 days after inoculation and from swabs at 1–4 days postinoculation

		Titer (log $TCID_{50}$ per g tissue or mL transport medium)*																						
		Tufted duck no.			Pochard no.				Mallard no.				Teal no.				Wigeon no.				Gadwall no.			
System	Organ	1	2	3	1	2	3	4	1	2	3	4	1	2	3	4	1	2	3	4	1	2	3	4
Nervous	Brain	4.8	3.5	5.2			3.8														1.5	1.5		
Respiratory	Trachea	3.8	3.2	4.5			4.2	0.8			3.2	2.5									1.5	4.5	2.8	
	Lung	3.8	2.5	5.5			3.5			1.2	1.5					3.2	0.8					0.8	1.8	
	Air sac	2.8	2.2	3.5			3.5					1.2					2.2		0.8	2.5	1.5	4.5	3.2	
Digestive	Pancreas	6.2	2.5	4.8			5.8	1.5								2.5						1.8		
	Liver	6.2		0.8			3.8													1.2		2.2		
	Jejunum	2.5		1.8			2.8																1.2	
	Colon	3.5		1.5			3.5										1.5		1.5				3.5	
Other	Spleen	5.5	0.8	0.8	0.8		1.5										1.5						2.8	
	Kidney	2.8	1.2	2.2			3.5			0.8							1.2						2.2	
Swab	Cloaca, 1 dpi																							
	Cloaca, 2 dpi						1.8																	
	Cloaca, 3 dpi						1.8																	
	Cloaca, 4 dpi																							
	Pharynx, 1 dpi	4.2	3.8	4.8			3.8	3.2	3.2	2.8	4.5	3.2			1.8	1.8	3.5		2.8		3.5		4.2	2.8
	Pharynx, 2 dpi	4.8	5.8	5.8	1.8	2.8	6.2	2.2	3.2	3.2	3.5	2.5				2.8			2.2					
	Pharynx, 3 dpi	4.5	5.2	5.2			5.5	2.5	2.8	2.5	2.5	1.8										1.8		
	Pharynx, 4 dpi	4.5	4.2	3.8			4.8	2.5	1.6	2.2											1.8		1.8	

*$TCID_{50}$, median tissue culture infectious dose; white, no virus isolated; yellow, virus titer 0.8–1.9; orange, 2.0–3.9; red, 4.0–5.9; dark red, 6.0–6.2; dpi, days postinoculation.
