# Supplementary material for: Wild Ducks as Long-Distance Vectors of Highly Pathogenic Avian Influenza Virus (H5N1)
Source: Emerg Infect Dis. 2008 Apr;14(4):600–7. doi: 10.3201/eid1404.071016 (PMC2570914; doi:10.3201/eid1404.071016)
Supplement: Appendix Table 4 — Detection of highly pathogenic avian influenza virus (H5N1) by reverse transcription-PCR from organs of wild ducks at 4 days postinoculation and from swabs at 1-4 days postinoculation [file 07-1016_appT4.pdf]

Appendix Table 4. Detection of highly pathogenic avian influenza virus (H5N1) by reverse transcription–PCR from organs of wild ducks at 4 days postinoculation and from swabs at 1–4 days postinoculation

		Cycle threshold value*																						
		Tufted duck no.			Pochard no.				Mallard no.				Teal no.				Wigeon no.				Gadwall no.			
System	Organ	1	2	3	1	2	3	4	1	2	3	4	1	2	3	4	1	2	3	4	1	2	3	4
Nervous	Brain	14	15	14	36		15	24			34					37			34		35	20	34	
Respiratory	Trachea	16	24	21		34	20	23	32	28	23	23	30		30	31	35	32	30	29		22	22	35
	Lung	18	26	16	32	35	24	31	30	22	26	26	29		30	23	28	31	31	29	25	21	26	36
	Air sac	18	26	23		38	20	27	26	24	28	29	25	nd	26	25	24	27		26		22	26	36
Digestive	Pancreas	15	26	22			18	22	33	37	27	36	35			26	35		29			24	27	
	Liver	14	34	32			18	37		37	33	37			35	37	34	35	31	39	28	16	28	39
	Jejunum	23		28			17			28	30	29			31		27		24	30	30	23	30	
	Colon	21	30	27			21	30		31	29	31	32		32	32	24	31	26	33		25	22	
Other	Spleen	18	35	26			35	27	28	27	19	34	36			35	30	34	31	32		24	25	
	Kidney	21	29	24			19	31	33	25	18	27	32		33	27	31	33	30	34		28	26	38
Swab	Cloaca, 1 dpi				33						31													
	Cloaca, 2 dpi	32		35			27	30														34		
	Cloaca, 3 dpi	30					28				32													
	Cloaca, 4 dpi	32	33				28																	
	Pharynx, 1 dpi	22	26	21	31	28	23	27	26	24	21	26	30		28	31	26	31	30		29		25	31
	Pharynx, 2 dpi	19	20	18	31	25	20	28	30	25	27	30	34		30	28	32		26		35		32	37
	Pharynx, 3 dpi	22	20	20	32	29	20	24	26	26	27	30				33	35		34		38	33	34	
	Pharynx, 4 dpi	22	23	24	34	33	21	25	30	29	31	36			38	37					33	30	34	

*White, no virus detected; yellow, 30–39 Ct; orange, 20–29 Ct; red, 10–19 Ct; dpi, days postinoculation.
