# Supplementary figures and images for: Wild Ducks as Long-Distance Vectors of Highly Pathogenic Avian Influenza Virus (H5N1)
Source: Emerg Infect Dis. 2008 Apr;14(4):600–7. doi: 10.3201/eid1404.071016 (PMC2570914; doi:10.3201/eid1404.071016)

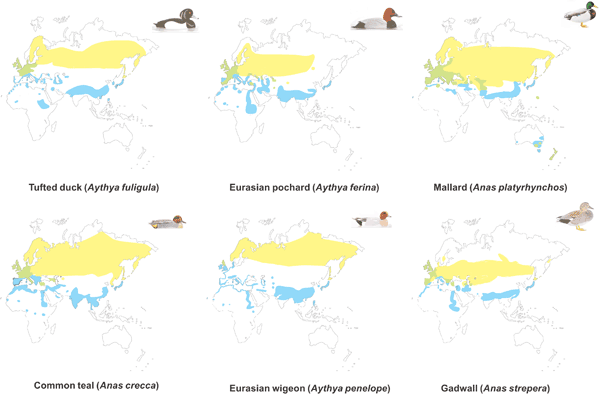

Supplement: Appendix Figure 1 — Distribution in the eastern hemisphere of the 6 wild duck species used in this study. Yellow, summer (breeding) range; blue: winter range; green, permanent range. (Sources: del Hoyo J, Elliot A, Sargatal J, editors. Handbook of the birds of the world. Volume 1: Ostrich to ducks. Barcelona: Lynx Edicions, 1992; Mullarney K, Svensson L, Zetterström D, Grant PJ. ANWB bird guide of Europe [in Dutch]. Baarn, the Netherlands: Tirion Uitgevers, 2000.) [file 07-1016_app1.gif]

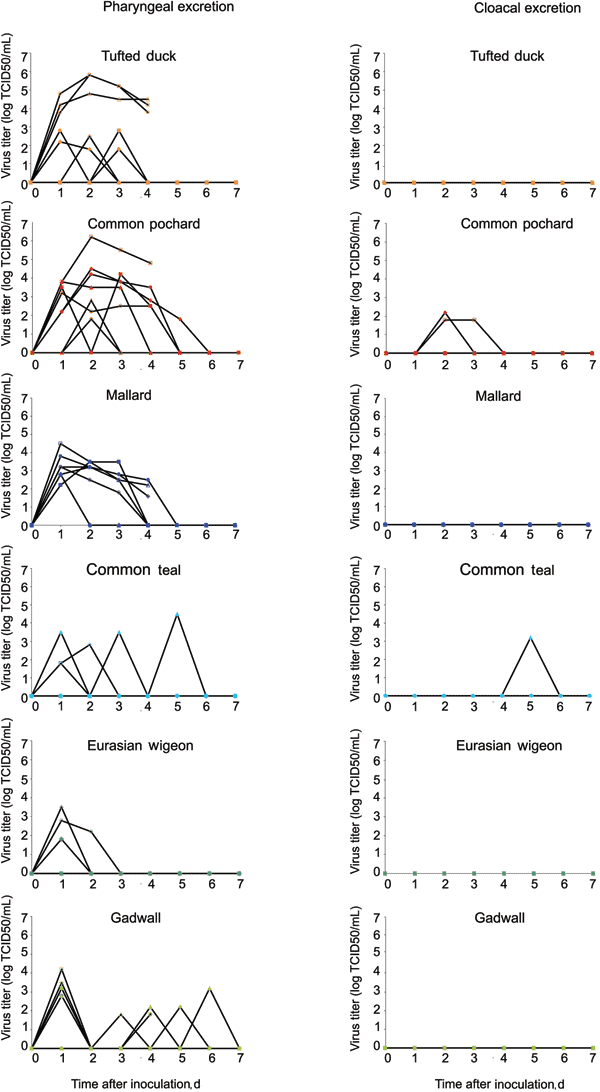

Supplement: Appendix Figure 2 — Individual pharyngeal (A) and cloacal (B) excretion of highly pathogenic avian influenza virus (H5N1) in wild duck species, by virus isolation. [file 07-1016_app2.gif]

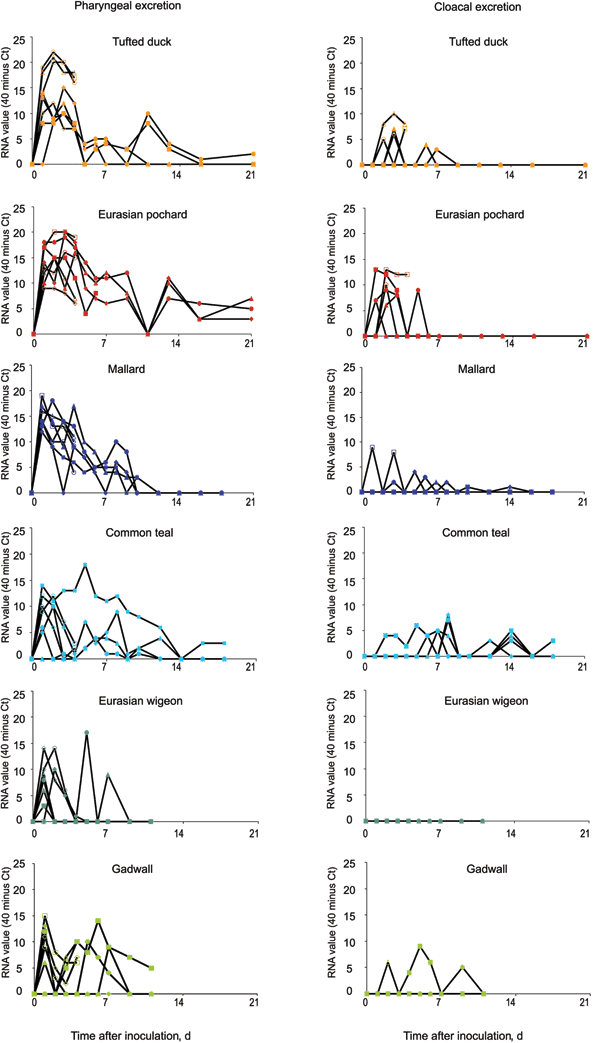

Supplement: Appendix Figure 3 — Individual pharyngeal (A) and cloacal (B) excretion of highly pathogenic avian influenza virus (H5N1) in wild duck species, by reverse transcription-PCR. [file 07-1016_app3.gif]

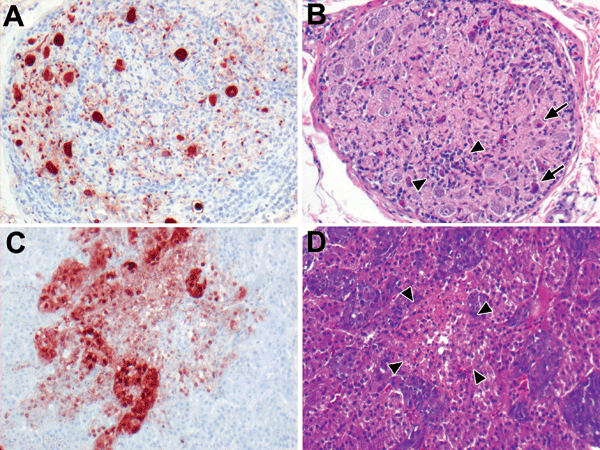

Supplement: Appendix Figure 4 — Highly pathogenic avian influenza virus (H5N1) infection in tufted ducks. A) Neurons and satellite cells in a mesenteric ganglion expressing abundant influenza virus antigen. B) Ganglioneuritis in the same mesenteric ganglion, characterized by neuronal necrosis (arrows) and lymphocyte infiltration (between arrowheads). C) Expression of influenza virus antigen in medullary and cortical cells of an adrenal gland and D) focal necrosis, characterized by hypereosinophilia, pyknosis, and vacuolization (between arrowheads). Original magnification ×100. Tissues were stained either by immunohistochemistry that used a monoclonal antibody against the nucleoprotein of influenza A virus as a primary antibody (A, C) or with hematoxylin and eosin (B, D). [file 07-1016_app4.gif]
